# Precisely Endowing Colloidal Particles with Silica Branches

**DOI:** 10.1038/s41598-019-44742-x

**Published:** 2019-06-13

**Authors:** Bin Zhao, Dongzhi Li, Yue Long, Kai Song

**Affiliations:** 1 Key Laboratory of Bio-Inspired Materials and Interfacial Science, Technical Institute of Physics and Chemistry, Chinese Academy of Sciences, 100190 Beijing, China; 2 Environmental Monitoring Station of Chenghua District of Chengdu, 610056 Chengdu, China

**Keywords:** Chemistry, Physical chemistry

## Abstract

A method to modify colloidal particles with silica rods in a water/*n*-pentanol system is reported here. Because of the interfacial tension between aqueous and *n*-pentanol phase, water which surrounds the colloidal particles de-wets into droplets during the deposition process of silica. As a result of unidirectional deposition, silica rods grow perpendicularly on the surface of the colloidal particles at the site of the smallest curvature where the water droplet has been de-wetted. By controlling the hydrolysis conditions, particles with certain number of branches or rambutan-like particles can be obtained. This approach opens a path towards the higher levels of colloidal complexity.

## Introduction

Branched particles, with multiple arms growing on core, have attracted considerable attention because of their unique electronic^1–4^, catalytic^5^, photophysical^6,7^, dispersion^8^ and self-assembling properties^9,10^, comparing with those traditional fibroid or spherical colloidal particles. Branching mechanisms varies with materials and synthesizing strategies including twinning, polymorphism, crystal splitting, and oriented attachment^11^. However, the material aspect of the reported branched particles are restricted to semiconductors^12,13^, metals^14–17^ or metal/semiconductor composites, where arms are formed by the epitaxial growth from the seed crystals^18–20^. Recently, crystal-like branched particles with mesoporous silica arms have been studied, its mesoporous property and partially controllable morphology makes it applicable in many areas^21–23^.

Silica coating, via the hydrolysis of tetraethoxysilane in ethanol/ammonia mixture, namely Stöber method, has been widely used in surface modification^24,25^. This strategy endows colloidal particles with manipulated interaction potentials, additional surface functionalities, and possibility of introducing fluorescent dyes to the surface^26,27^. Colloidal particles could preserve their original shape when coated with a thin layer^28^, or become rounded if the coating layer is thick^29^.

Despite the few successful examples which are based on the predetermined geometry of the seed particles, to anisotropically modify the shape of particles via selective silica coating still remains challenging at the current stage^30–33^. Recently, Zhang *et al*. and Kuijk *et al*. developed a one-pot strategy to form anisotropic nanostructures through unidirectional growth of silica rods^34,35^. In a PVP/Na3Cit stabilized water/*n*-pentanol emulsion, hydrolyzed TEOS diffuses from *n*-pentanol into water droplet and deposit silica from within. Due to anisotropic supply of reactants, silica rods only grow to one side; hence, forming a one-dimensional structure. Later, Nie, *et al*. synthesized amphiphilic silica rods with two segmented components by adding two different silica precursors in turn^36^. As a step further, colloidal particles of various materials were connected with silica rods via unidirectional silica deposition, which resulted in matchstick-like composite rods^37^. Also, by attaching PMMA bulb to silica rods, matchstick-like and hedgehog-like particles were prepared^38^. And silica rods have also been grown on the surface of colloidal particles via anisotropic hydrolysis of silica precursor, resulting in urchin-like morphologies^39,40^. All the above methods enriched the library of silica-based colloidal particles. However, it is still highly desirable to anisotropically and precisely modify colloidal particles with silica rods.

Here we reported a revised strategy to modify the shape of colloidal particles by precisely endowing them with directional rods, where silica rods can be selectively grown on the specific part of the particle. Non-spherical shaped colloidal particles including cubes, dumbbells, and discs were chosen as seed particles. The silica rods grew preferentially at the sites of the smallest curvature, while the morphology of the rod can be tuned.

## Results and Discussion

**Figure 1:**
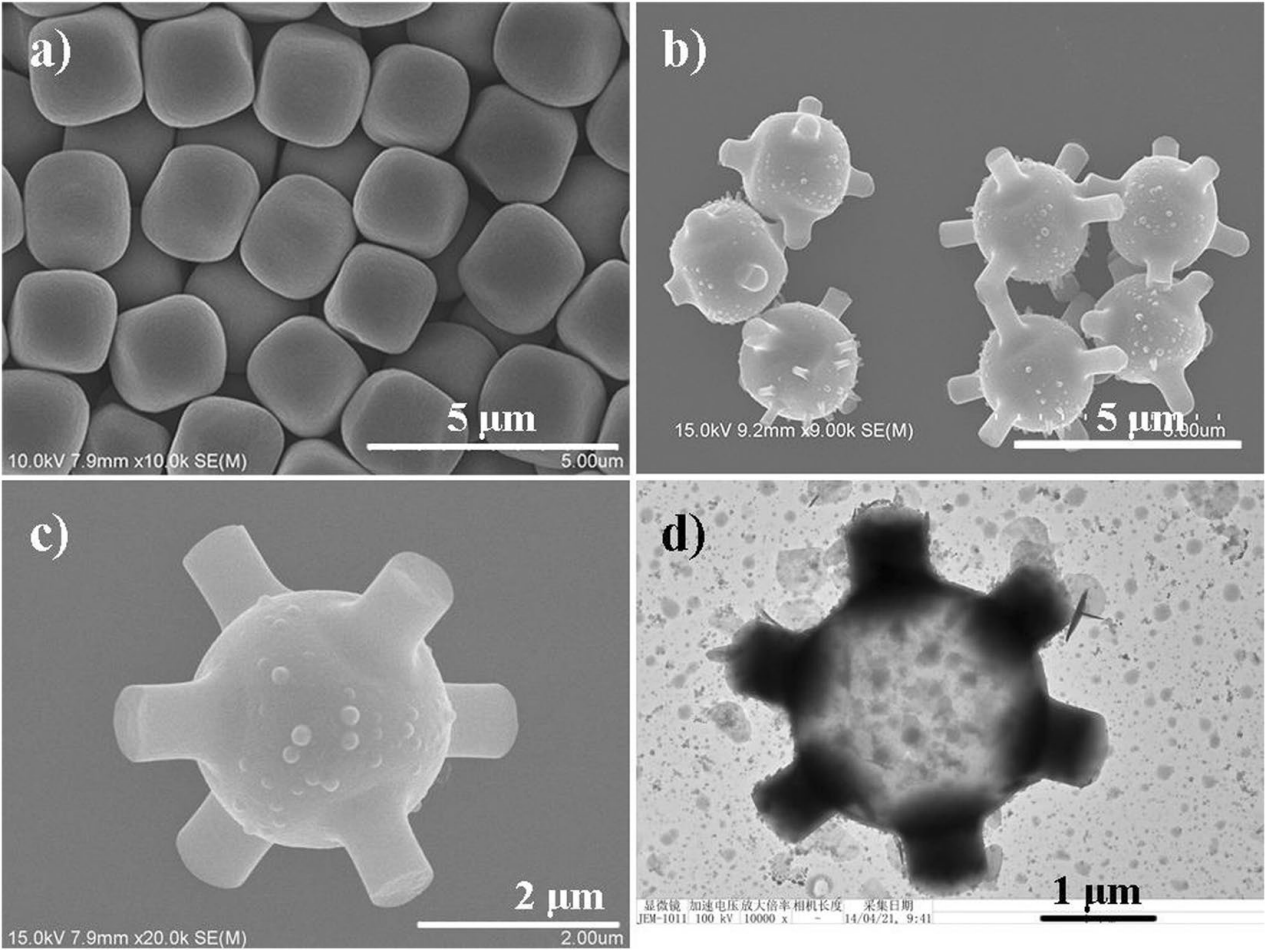
SEM images of (**a**) Bare α-Fe_2_O_3_ cubes, (**b**) SiO_2_ rod modified α-Fe_2_O_3_ cubes, showing the uniformity of branched morphology, (**c**) Amplified SEM image of a branched cubic particle, (**d**) TEM image of a HCl-etched branched particle.

Site specific growth of silica rods on hematite particles was realized in a two-stage process (see Supporting Information for details). Firstly, hematite microcubes were synthesized following a previously reported method and used as the seed particle (Fig. 1a)^41^. In the subsequent step, the prepared cubic particles were dispersed in a solution of polyvinylpyrrolidone (PVP) in *n*-pentanol, and sonicated for 2 h before adding ethanol, water, sodium citrate (Na_3_Cit) and aqueous ammonia to the dispersion. After shaking, a water-in-*n*-pentanol emulsion was formed. The resulted water droplets were attached to the surface of the seed particles and formed a thin layer. Upon the addition of TEOS, hydrolysis and condensation occurred in water which was attached to the particle surface to grow silica rod on it. In the end, six silica rods with each positioned perpendicularly at the center of the plane were obtained (Fig. 1b). An enlarged SEM image is shown in Fig. 1c to inspect the surface morphology of the branched particle. As can be seen that the surface of the silica rod is relatively smooth, and the main body is quite rough. Figure 1d shows a TEM image (Fig. 1d) of the branched particle after removing the hematite core by HCl etching, in this way, the distribution of silica can be examined. A continuous layer of silica is obtained, to which six silica rods was found attached on the surface.

**Figure 2:**
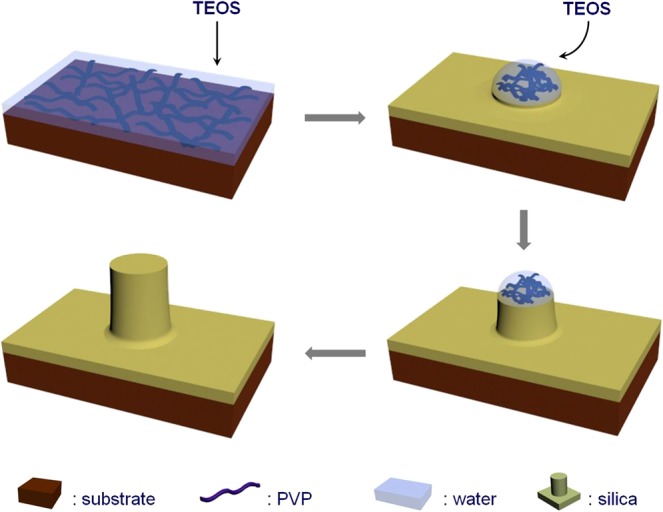
Schematic illustration of the growth mechanism of silica rods on the surface of the particles. In step 1, PVP molecules absorbs onto the seed particles, after which water is added; in step 2, water spread to form a thin layer on the surface of the particle, then TEOS is added; in step 3, after the formation of uniform silica shell, semi-spherical shaped water droplet emerges due to de-wetting effect; in step 4, unidirectional deposition of silica through water droplet resulted in the growth of silica rod perpendicular to the surface of the particle; in step 5, silica rod is formed when hydrolysis is stopped.

A possible mechanism of the rod growth is shown in Fig. 2. After the cubic particle was added into the PVP in *n*-pentanol solution, some of the PVP molecules absorbed to the surface of the cube and formed a dense layer as described elsewhere^25^. Following the addition of water and emulsification, a water-in-*n*-pentanol emulsion was formed. Due to the strong affinity between water and PVP, water droplets were drawn to the dense PVP layer at the surface of the cube and spread to form a layer surround the particle (see Fig. S1)^42,43^. This process, so-called spreading, is a typical phase separation occurs at the solid-liquid interface, and is determined by spreading coefficient S^44^. Before hydrolysis, a thin layer of water was formed surround the particle due to the surface PVP layer, and S > 0. After the addition of TEOS, hydrolysis occurred at the water layer, and a uniform shell of silica was formed on the surface of the seed particles (evidenced by the SEM image of the HCl-etched hollow silica shell shown in Figs 1d and S3). The original solid particle surface was then replaced by the deposited silica, and de-wetting occurred when S < 0. Consequently, the thin layer of water transformed to a semi-spherical droplet attaching on the silica shell (Fig. S2). It is noteworthy that water in this system refers to the highly concentrated PVP-bound water, which is also called “bound water”. De-wetting was found to not occur without the existent of the highly concentrated PVP as studied by Zhang *et al*.^43^. Subsequently, anisotropic silica deposition took place in the water droplets and resulted in epitaxial growth of the silica rods as previously described^35^.

It is interesting to find that the growth site of the silica rods was curvature-dependent. Walker *et al*.^45^. once reported geometric curvature controls the chemical patchiness on Au colloidal particles because regions of the particle surface with different curvature occupies varied surface density of the ligands’ head groups, which become charged at different pH values of the surrounding solution for site-selectively chemical patchiness. Furthermore, Xia *et al*.^46^ presented a quantitative analysis of the role played by PVP in seed-mediated growth of Ag nanocrystals. In their study, for the cubic colloids, the corners and the faces occupies different concentration of PVP molecule. That is, the smaller the curvature, the higher surface density of PVP molecules. Similarly in our system, the bound water surrounded by the colloidal particles started to de-wet after a uniform silica layer was formed, they chose the location with smaller curvature and higher density of PVP molecules. In the case of cubes, since faces possess smaller curvatures compare to the edges and vertices, the water droplet located at the center of the face in order to maintain the minimum energy of the system. The de-wetting process of water from thin layer to semi-spherical droplet is determined by multiple parameters including the amount of water, size and morphology of the particles. Furthermore, the silica rods shown in Fig. 1b have two types of ends, flat and round. This is dependent on whether the water droplet is completely consumed in the hydrolysis. As proposed by Kuijk *et al*., for the rods with flat ends, the water droplet is still attached to the end of the rod when the reaction stopped^35^. In the condition that water is completely consumed, silica rods stopped growing and obtained round ends.

**Figure 3:**
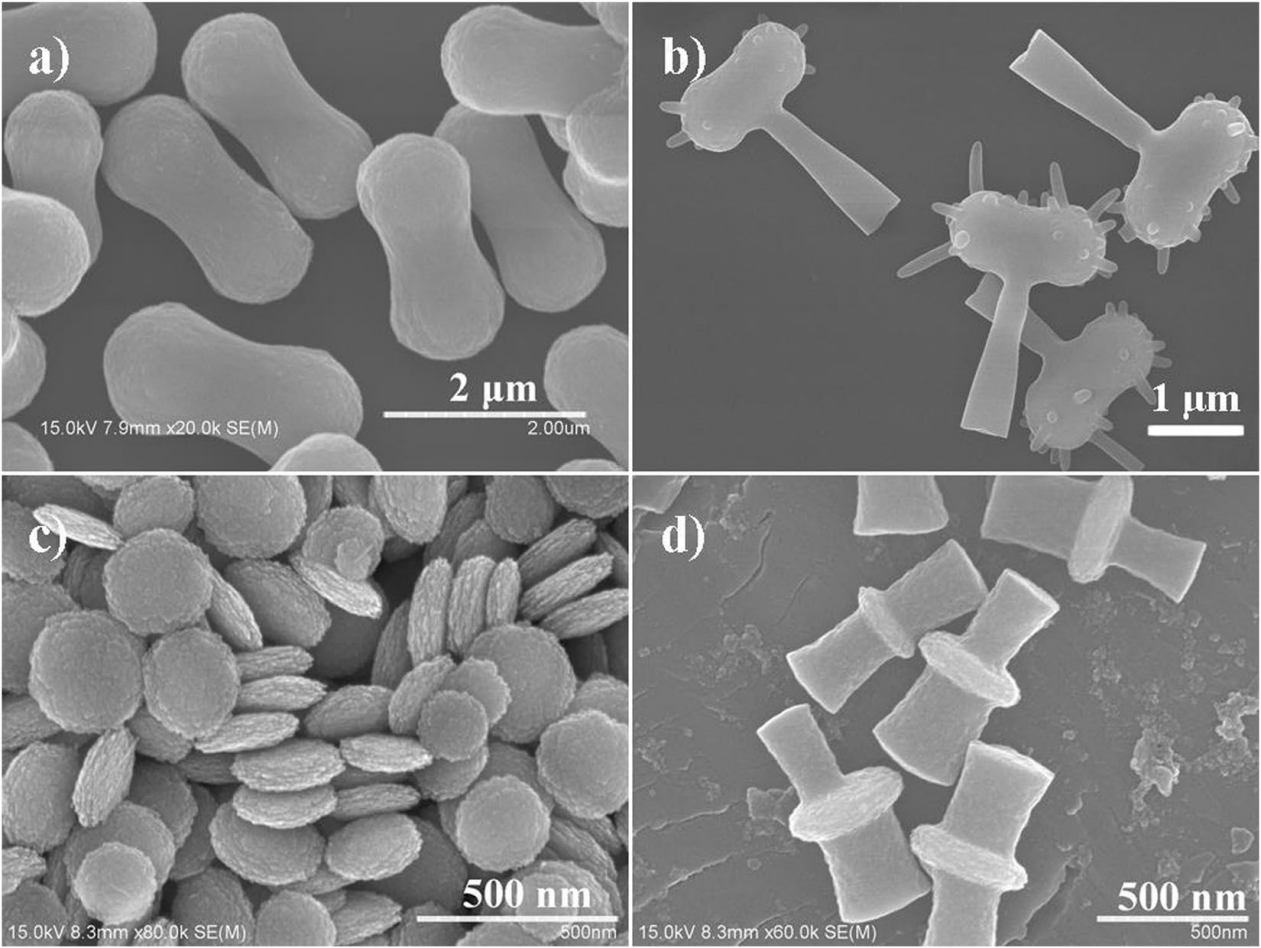
SEM images of (**a**,**b**) dumbbell-shaped particles before and after modification; (**c**,**d**) disc-shaped particles before and after silica rods modification. Silica rods grow at the site of the smallest curvature.

In order to verify our hypothesis of curvature-based SiO_2_ rod growth, two other hematite colloidal particles with the shape of dumbbell and disc were utilized as the seed particles^47,48^. According to the curvature dependent mechanism, silica rods should site-selectively grow on the area that possesses the smallest curvature of the particle, i.e. neck of the dumbbell and plane of the disc colloids. It can be seen in Fig. 3 the respective structures of the two colloids before and after the modification. Results show that the growth of silica rods matches well with our hypothesis. However, being different from cubes and discs, only one main silica rod was grown at the neck of the dumbbells, while a few smaller silica rods grew at the two tips. It was speculated that during the rod formation process, water droplets tends to set at the neck of the particle which possess the smallest curvature (Fig. S4); however, droplets at two tips are too far apart from the neck, hence are difficult to coalesce into a single droplet at the neck during the de-wetting process. These remote and isolated water droplets resulted in the formation of a few much smaller silica rods during the hydrolysis. In the case of disc-like particles, coalescence of water droplets at the same side give rise to the growth of one silica rod at each side of the disc. One more possibible reason behind the far seperated location of the silica rods is the repulsive electrostatic force between the silica branches, while the force should be generated by the citrate molecules and silanol groups on the surface of the grown silica rods.

**Figure 4:**
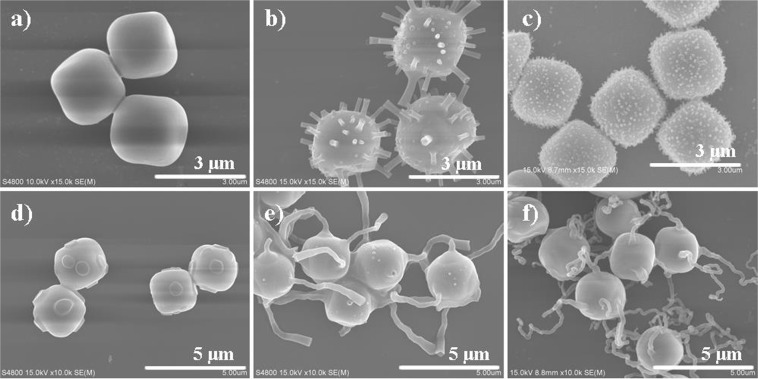
SEM images of the modified colloids with varied amount of water. (**a**) 1.0 mL, (**b**) 0.5 mL and (**c**) 0.2 mL, while the amount of aqueous ammonia is 0.675 mL; SEM images of the modified colloids with varied amount of aqueous ammonia: (**d**) 1.0 mL, (**e**) 0.23 mL and (**f**) 0.055 mL, while the total amount of water (including water in NH_3_·H_2_O) is 1.17 mL. Less water results in dense and smaller silica rods, and less ammonia results in longer, thinner and winding silica wires.

Furthermore, the morphology of silica-modified particles can be tuned by adjusting the parameters of hydrolysis. When keeping the concentration of ammonia constant, increasing the amount of water to 1.0 mL resulted in spherical particles with uniform silica coating as shown in Fig. 4a; whilst, decreasing the water content (0.5 and 0.2 mL) led to multiple rods growing on the particle surface forming rambutan-like structures (Fig. 4b,c). In the former case, the concentration of PVP decreases with increasing the amount of water in the system, and consequently reduce the interfacial tension as was found in previous studies^22^. As a result, there is no coalescence of water and uniform silica coating was obtained. Similar phenomenon is also observed in the system of pure silica rod^35^. And in the case of less water, the rough surface of hematite may play a key role in the generation of multiple rods (as explained in Fig. S5). As the diameter of SiO_2_ rods is determined by the size of water droplets, only multiple small SiO_2_ rods were formed with the decreased water content due to the formation of tiny water droplets on particle surface (Fig. 4b), and the rods became even smaller with the further reduction of water (Fig. 4c). When keeping the concentration of water constant, increasing the concentration of ammonia led to the formation of shorter silica rods (Fig. 4d). Contrastingly, as the concentration of ammonia decreased, the six straight and thick silica rods transformed into longer, thinner and winding wires. Hollow silica tubes were obtained with the further reduction of the concentration of ammonia (Fig. S6). The decreased ammonia concentration resulted in slow hydrolysis of TEOS, thus, more silica were deposited on the surface rather than at the center of the droplets. The change of rods to wires was the result of the altered interfacial tension. The role of ethanol cannot be ignored either, it smears out the interface between water and *n*-pentanol. Only spherical products were obtained with large amount of ethanol, and no reaction took place without ethanol.

**Figure 5:**
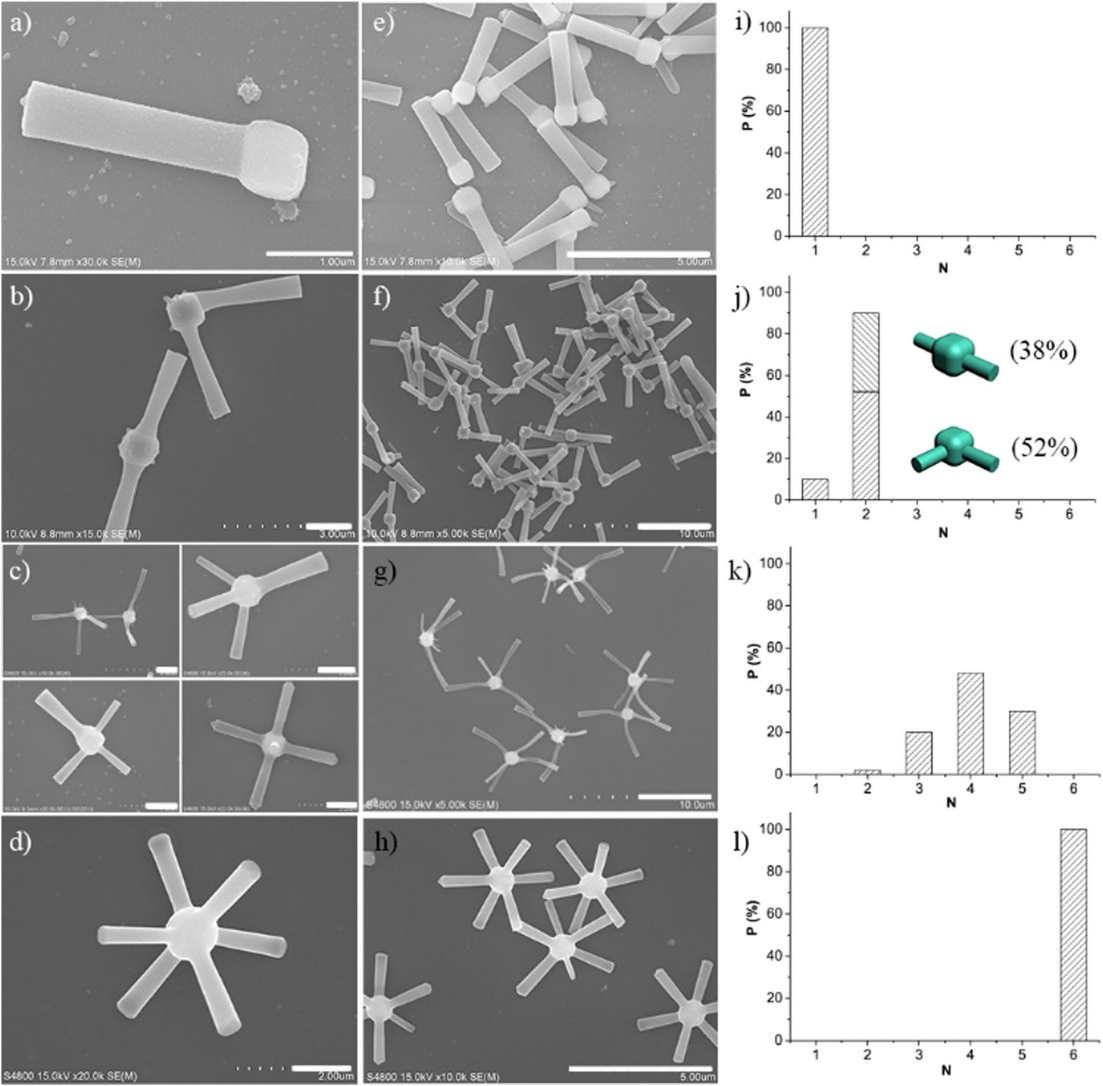
Typical SEM images of the modified colloids with varied branches. Scale bars: (**a**–**d**), 1 μm; (**e**–**h**), 5 μm. (**i**–**l**) Statistic calculation of the purity of the modified colloids with varied branches.

Recently, Wiesner *et al*. proposed an epitaxial growth mechanism to synthesis branched multi-compartment silica for mimicking colloids with valence^22^. However, this strategy is limited to silica particles. Moreover, the obtained product contains a mixture of one, two, three and four branched particles, but neither can be separated from others. In our approach, it is possible to selectively obtain particles with exact number of branches by controlling the amount of water and ammonia used in the fabrication process. As shown in Figs 5a and S7a, when the total amount of water used was 1.382 mL (including the water in aqueous ammonia) and the concentration of ammonia was 11.07 wt%, unitary mono-branched particles were obtained. Decreasing the amount of water to 1.300 mL and increasing the concentration of ammonia to 14.22%, the particles with two branches became the majority, counted as 90% of the total products. Interestingly, for di-branched cubic particles, there are two “geometric isomers”, in this case, 58% of ortho-isomer and 42% of para-isomer were obtained (Figs 5b and S7b). Further decreasing the amount of water and increasing the concentration of ammonia, a mixture of particles with three, four and five branches were obtained (Fig. S7c). For this mixture, it was difficult to get the pure product with exact number of branches. As shown in Fig. 5c, there are two geometric isomers for the particles with three branches and two isomers for four branched one. For particles with five (Fig. S7c) and six branches (Figs 5d and S7d), there is only one structure. Pure six branched particles can also be prepared as discussed above. Figure 5e–h are corresponding to a-d, but with a smaller magnification. Figure i–l are the statistic calculation of the modified colloids with varied branches.

In addition, it is noteworthy that tunable branched particles were only obtained from relatively smaller cubic seeds. In the experiment above, the edge length of the cube was 800 nm. When increasing the length of the edge to 2 μm, only spherical uniform silica coating or six branched particle were obtained. The exact mechanism of the effect of the water and ammonia on the number of branches is not exactly clear yet. As a possible rational, the explanation has to do with the energetics and the kinetics. For smaller cubic seed particles, water layer should coalescenc into a single big droplet to minimize the energy of the whole system during the de-wetting process. However, decreasing the amount of water resulted in higher concentration of PVP in the water, and increasing the concentration of ammonia resulted in faster hydrolysis. Reasonablly, this faster hydrolysis giving rise to the growning of silica rods before the bound water droplets converges to a big one, instead, it resulted in separated droplets at different faces of the cube. It is believed that the inhomogeneity of different cubes resulted in different geometric isomers. And for bigger cubic particles, e.g. 2 μm, it is more difficult for the mass transport get across the edge, thus producing unitary particles with six branches. Though the mechanism is still open for further investigation, the present result indicates a new route to endow seed particles with certain “valence”.

Here, iron oxides were chosen as the seed particle because of its magnetic separation ability. However, due to the extensive surface modification ability of PVP molecules, this method can be applied to a wide range of colloids of various materials and shapes (Fig. S8), and separation can be achieved by the widely used density gradient centrifugation method. Therefore, it is a general and facile method to obtain complex and anisotropic architectures, which can break the limitation of certain materials that possess highly symmetric geometries^9,49^.

## Conclusions

In summary, we have demonstrated a revised method to anisotropically modify colloidal particles with silica rods. The water layer bound by the PVP adsorbed on particle surface resulted in a uniform silica shell, and the water droplets de-wetted at the sites with the smallest curvature of the particle formed silica rods. In addition, the morphology of silica rods can be easily tuned by changing the hydrolysis conditions. The rambutan-like particles can be used in catalyst support or cell adhesion for their enhanced surface area and special branched morphology^50,51^. More importantly, because of the uniform size and complex structure, these silica-rod-modified branched colloidal particles are expected to find use as building blocks to fabricate new structures and devices. For example, the planar terminal of silica rods on the short silica-rod-modified particles tends to attach to each other (Fig. S9), and steric effect may dominate the assembly behaviors of long silica-rod-modified particles, similar to what has been observed on other branched particles^9^.

## Experimental Section

In a typical process, PVP (3 g) was dissolved in *n*-pentanol (≧99%, 30 mL) by sonication for 1 h. When PVP was dissolved, a dispersion of hematite cubes in water (20% w/v, 50 μL) was added, and sonicated for a further 2 h before adding a mixture of ethanol (3 mL), deionized water (840 μL) and aqueous solution of sodium citrate dihydrate (0.18 M, 200 μL). The resulting mixture was shaken by hand for 5 min to form a water/*n*-pentanol emulsion. Aqueous ammonia (28%, 675 μL) was then added and the flask was shaken for another 3 min before adding TEOS (≧98%, 500 μL) to the emulsion. After further shaking for 1 min, the flask was left to rest and the reaction was allowed to proceed overnight. Next, the resulted mixture was centrifuged 3 times with water and ethanol (1:1) before being transferred into water. The modification process of the dumbbell and disc shaped colloidal seeds are the same as the cubes.

## Supplementary information

Precisely Endowing Colloidal Particles with Silica Branches
