# Supplementary material for: Precisely Endowing Colloidal Particles with Silica Branches
Source: Sci Rep. 2019 Jun 13;9:8591. doi: 10.1038/s41598-019-44742-x (PMC6565735; doi:10.1038/s41598-019-44742-x)
Supplement: Supplementary file 1 — Precisely Endowing Colloidal Particles with Silica Branches [file 41598_2019_44742_MOESM1_ESM.pdf]

Supporting Information

Precisely Endowing Colloidal Particles with Silica Branches

Bin Zhao,[a,b] Dongzhi Li,[a] Yue Long,[a] and Kai Song*[a]*

[a]Laboratory of Bio-Inspired Smart Interface Science, Technical Institute of Physics and Chemistry, Chinese Academy of Sciences, Beijing 100190, China
E-mail: longyue@mail.ipc.ac.cn; songkai@mail.ipc.ac.cn
[b]Environmental Monitoring Station of Chenghua District of Chengdu, Chengdu 610056, China

1. Materials

Tetraethyl orthosilicate (TEOS) and ammonia (28%) were purchased from Alfa Aesar. Polyvinylpyrrolidone (PVP, Mw. 40k) was purchased from Sigma Aldrich. *n*-Pentanol (⩾ 99%) was purchased from Acros. Other chemicals were purchased from Sinopharm Chemical Reagent Co. Ltd. All of the reagents were of analytical grade and used without further purification. The water used throughout all the experiments was purified with the Millipore system.

2. Synthesis of seed particles

2.1 Hematite dumbbell-shaped and cubic colloids: The synthesis of hematite colloidal particles with different shapes was achieved by a process based on a method developed by Sugimoto et.al.[1] In a typical synthesis of dumbbell-shaped hematite colloidal particles, $FeCl_3$ (2 M, 100 mL) was firstly added to NaOH solution (90 mL, 6 M) in a 250 mL Pyrex bottle and agitated for 5 min followed by the addition of Na_2SO_4 (10 mL, 0.6 M), and agitation was continued for an additional 10 min. $Fe(OH)_3$ gel in a tightly sealed bottle, typically containing $Fe(OH)_3$ (0.9 M), Fe^{3+} (0.1

M), and SO_4^{2-} (30 mM) was placed in an oven preheated to 100 °C, and aged for 8 days. After the treatment, red products were collected by filtration and washed three times with deionized water and ethanol (1:1) before drying at 50 °C overnight. The procedure of the synthesis of hematite cubic particles was similar to the preparation of the dumbbell-shaped particles except the absence of Na_2SO_4.

Reference 1. Sugimoto, T., Khan, M. M., Maramatsu, A. & Itoh, H. Formation mechanism of monodisperse peanut-type α-Fe_2O_3 particles from condensed ferric hydroxide gel. *Coll. Surf. A* **79**, 233-247 (1993).

2.2 Hematite discs: Disc-shaped particles were fabricated using a method reported by Song et.al.[2] Iron chloride (0.75 mmol) was dissolved in deionized water (30 mL) as solution A. Sodium silicate (0.375 mmol) was homogeneously dispersed in deionized water (20 mL) as solution B. Both solutions were prepared at room temperature. The two solutions were then mixed to form a transparent orange colored solution, which was transferred into an autoclave (70 mL) and heated for 24 h at 140 °C. The red product was collected by centrifugation and rinsed with deionized water for several times. Finally, the product was dried in oven at 80 °C overnight. Analysis was performed with a scanning electron microscopy (SEM, Hitachi S-4800 at 15 kV).

Reference 2. Qu, J., Yu, Y., Cao, C.-Y. & Song, W.-G. α-Fe2O3 nanodisks: layered structure, growth mechanism, and enhanced photocatalytic property. *Chem. Eur. J.* **19**, 11172-11177 (2013).

3. Modifying colloidal particles with silica rods

In a typical process, PVP (3g) was dissolved in *n*-pentanol (⩾ 99%, 30 mL) by sonication for 1 h. When PVP was dissolved, a dispersion of hematite cubes in water (20% w/v, 50 μL) was added, and sonicated for a further 2 h before adding a mixture of ethanol (3 mL), deionized water (840 μL) and aqueous solution of sodium citrate dihydrate (0.18 M, 200 μL). The resulting mixture was shaken by hand for 5 min to form a water/*n*-pentanol emulsion. Ammonia (28%, 675 μL) was then added and the flask was shaken for another 3 min before adding TEOS (⩾ 98%, 500 μL) to the emulsion. After a further shaking for 1 min, the flask was left to rest and the reaction was allowed to proceed overnight. Next, the resulted mixture was centrifuged several times with water and ethanol (1:1) before being transferred into pure water. The modification process of the dumbbell- and disc-shaped colloidal seeds are the same as the modification of cubes.

Various numbers of branched particles were synthesized in a similar process except the amount of water and aqueous ammonia used in the process were varied. Here, 800 nm hematite cubes were used as the seed particles. In a wide range of total amount of water from 1.280 mL to 1.270 mL and the concentration of ammonia from 15.76 wt% to 16.48 wt%, a mixture of three, four and five branched particles were obtained. For the six branched particles, the amount of water and aqueous ammonia were 0.390 and 1.005 mL, respectively, and the corresponding total amount of water and the concentration of ammonia are 1.25 mL and 17 wt%, respectively.

Fluorescence labeling

A powerful and convenient tracer method is applied in identifying the wettability of one particle's different regions. Water-soluble CdTe quantum dots were chosen as a fluorescent probe.

To test its solubility, a water in n-pantanol emulsion was prepared, to which CdTe quantum dots were added. It can be clearly seen in the confocal laser scanning microscope images (Figure S1a, b and c) that hydrophilic CdTe quantum dots with red emission (λ_{max}=680 nm) exsited in the water phase rather than in the *n*-pentanol phase. Therefore, it can be used to lable the water layer that surounds the hematite cubes as claimed. It can be seen in Figure S1 d, e, and f that the hematitie cubes were dyed continueously on the surface of the particle in the PVP/Na_3Cit stabilized water/*n*-pentanol system, which suggests that a water layer is formed and surrounded the hematite cube. If selectively removing PVP or water in the system, the hematite cubes were found aggregated and sunk to the bottom rather than dispersed in the mixture.

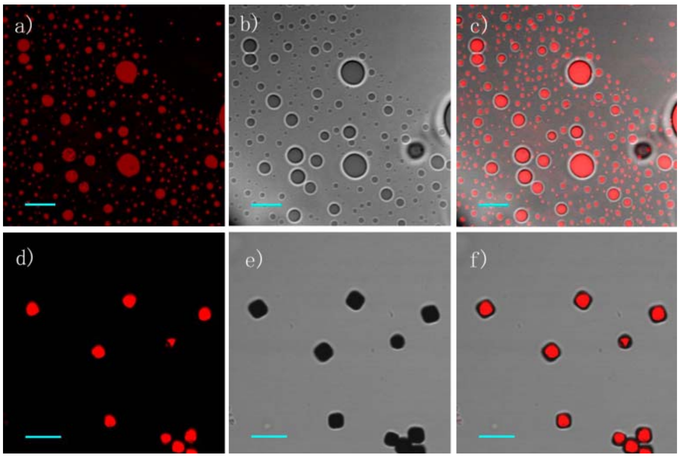

Figure S1. Confocal microscope images of hydrophilic TeCd quantum dots dispersed in water/*n*-pentanol emulsion a) dark field image, b) bright field image and c) combination of both fields; TeCd quantum dots in the dispersion of hematite cubes in a PVP/Na3Cit stabilized water/*n*-pentanol emulsion d) dark field image, 3) bright field image and f) combination of both fields.

Cryo-TEM

The cryo-TEM samples were prepared in a controlled environment vitrification system (CEVS) at 25 °C. A micropipette was utilized to load 5 μL of product onto a TEM carbon grid, which was blotted with two pieces of filter paper, resulting in the formation of a thin film suspended on the mesh holes. After 5 s, the sample was quickly plunged into a reservoir of liquid ethane (cooled by nitrogen) at -165 °C. The vitrified sample was then stored in liquid nitrogen until being transferred to a cryogenic sample holder (Gatan 626) and examined with a FEI Tecnai 20 TEM (120 kv) at about -174 °C. The phase contrast was enhanced by underforce. Images were recorded on a Gatanmultiscan CCD and processed with a Digital Micrograph.

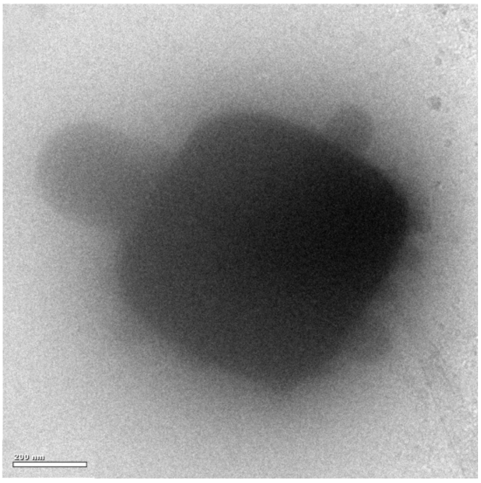

Figure S2. Cryo-TEM images of hematite cube during hydrolysis process: it can be clearly seen that a semi-spherical droplet attached to the surface of the cube after 30 min hydrolysis of TEOS. The absence of 6 rods on one particle should be due to the relative high concentration of seed particles for the cryo-TEM sampling.

4. Preparation of hollow silica shells from HCl-etching

To further illustrate the effect of the “bound” water in the formation of homogeneous silica coating, an aqueous HCl solution (6 M) was used to etch the hematite seeds, so that the silica shell can be clearly identified (Fig S2). When a relative large amount of water was used, the hematite cubes were modified with six silica rods with one on each face, at the same time, a layer of 70 nm silica coating was obtained. While reducing the amount of water, tiny silica rods was obtained on the surface of the cube and the thickness of the homogenous silica coating was reduced to 50 nm. At reduced water content, only small water droplets were formed on the particle surface, which gave rise to the formation of small silica rods after the hydrolysis.

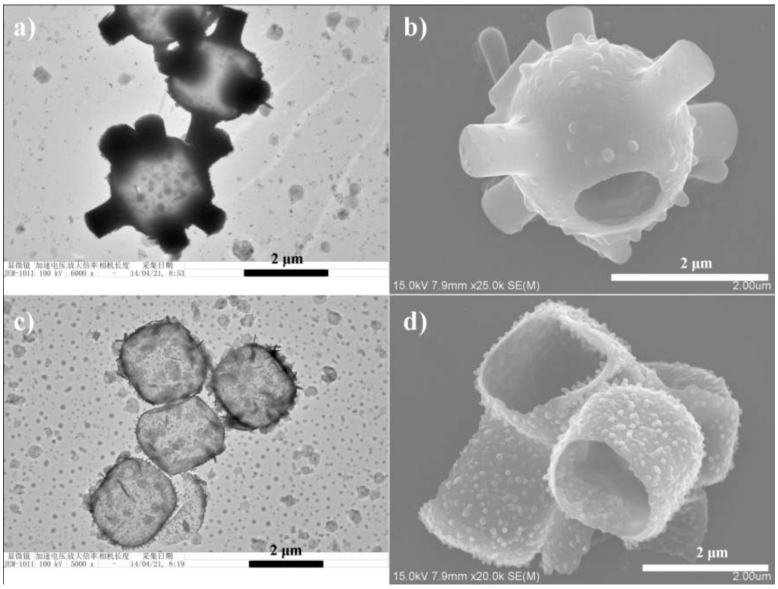

Figure S3. TEM (a) and SEM (b) images of the HCl-etched hollow silica shells with six silica rods obtained from high water concentration; TEM (c) and SEM (d) images of hollow silica shells with abundant tiny silica rods obtained from low water concentration.

5. Curvature calculation of the colloidal particles

Here the curvature calculated is the principle curvature of the particle. For non-spherical particles, the curvature of a surface at a given location is, in general,

defined as $\kappa=(1/R_1+1/R_2)/2$ (where R_1 and R_2 are the principal radii of the curvature)*. Taking the dumbbell hematite for example, the curvature at the approximately hemispherical tips of the micro-dumbbells is $\kappa_{tip}=1.68\mu m^{-1}$, and the curvature at the neck is $\kappa_{neck}= 0.74+ 0.03/-0.05\mu m^{-1}$, where one radius of curvature is negative.

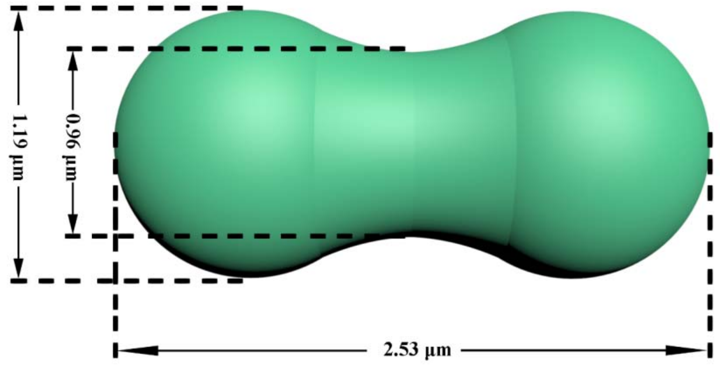

Figure S4. Schematic illustration of the curvature calculation of the dumbbell colloid used in the study.

6. The explanation on the generation of multiple silica rods in the case of less water

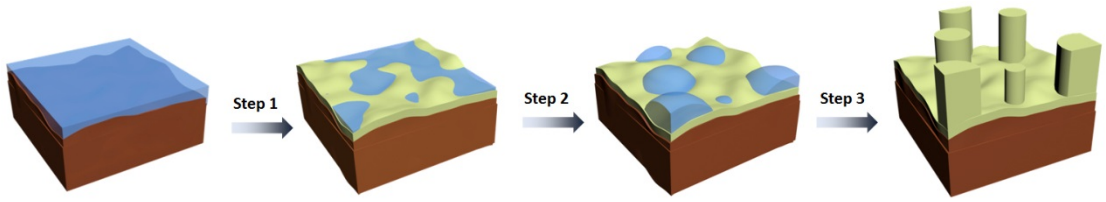

Figure S5. Schematic illustration of the proposed mechanism of the generation of multiple silica rods in the case of less water. when the amount of water decreased, the water layer surrounded the seeds became thinner. In step 1, similar to the rich water system, a silica shell was first formed in the hydrolysis step, which consumes a great potion of the absorbed “bound water”. Therefore, only some isolated smaller water droplets were formed in the subsequent de-wetting process (step 2). These isolated water droplets cannot coalesce into a single droplet but to form multiple smaller rods on the surface (step 3).

7. Silica tube modification with lower ammonia concentration

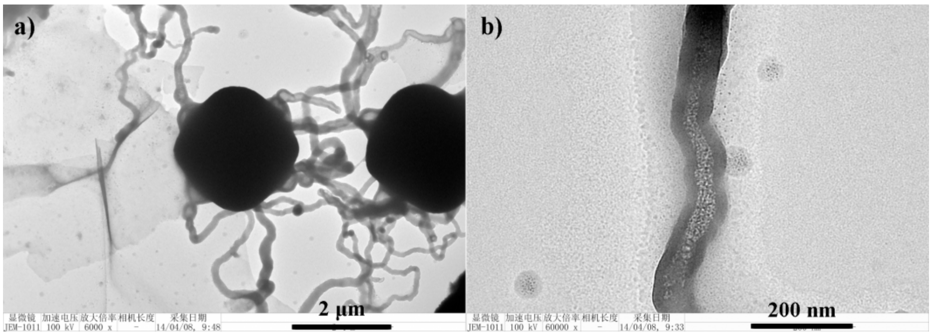

Figure S6. a) Silica tubes grown on the hematite cube with low concentration of ammonia, b) amplified image of a silica tube.

8. The tunable branched particles

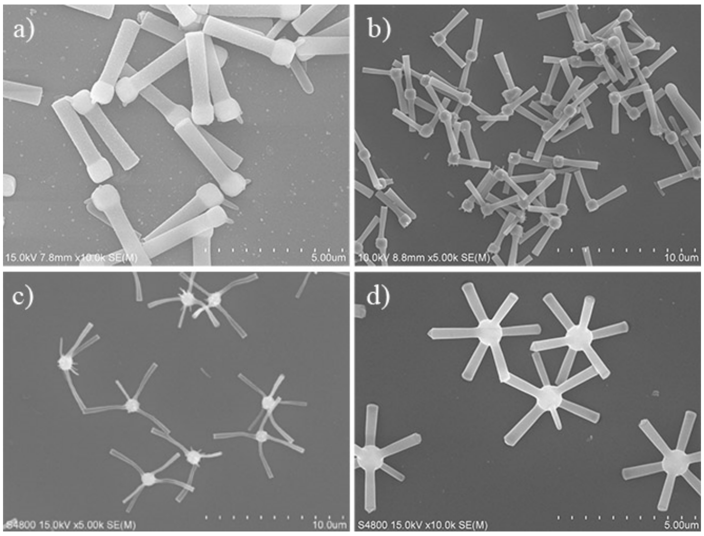

Figure S7. SEM images of particles with varied branches. a) one branch, b) two branches, c) three-five branches, d) six branches.

9. Modifying colloidal particles with different shapes and components

It is a general and facile method of obtaining complex and anisotropic architectures by this means, and a wide range of seed particles with different components and shapes have been modified with silica branches as shwon in Figure S7, including metals (Au), metal oxides (Fe_3O_4), non-metallic oxides (SiO_2), metal–

organic-frameworks (MOFs, Prussian blue), polymers (polystyrene (PS) and polydimethylsiloxane (PDMS) with morphologies such as cages (Au, hollow nanoporous nanoparticle), spheres (Fe_3O_4, SiO_2, PS), micro-boxes (Prussian blue), and even planes (PDMS).

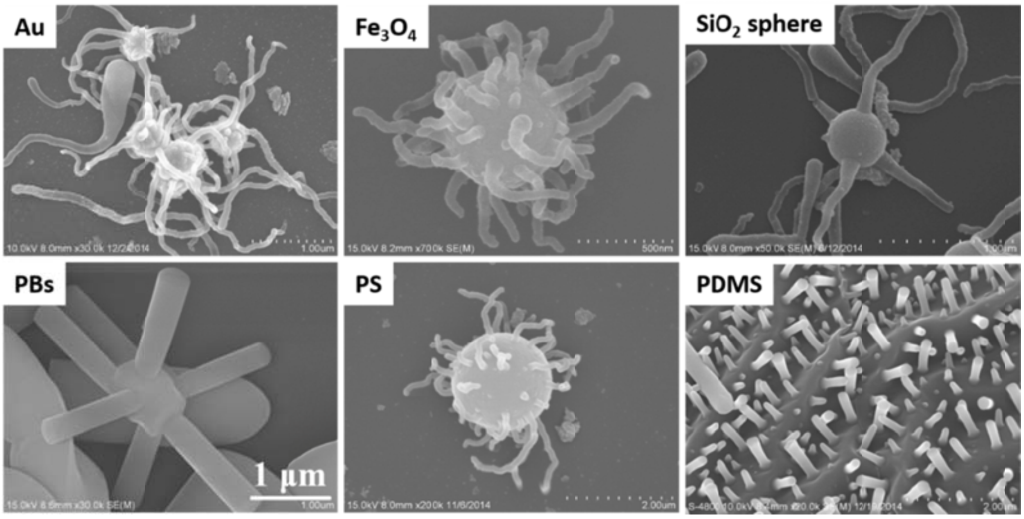

Figure S8. SEM image of silica modified seed colloids with varied materials and shapes.

10. Self-assembly of branched particles

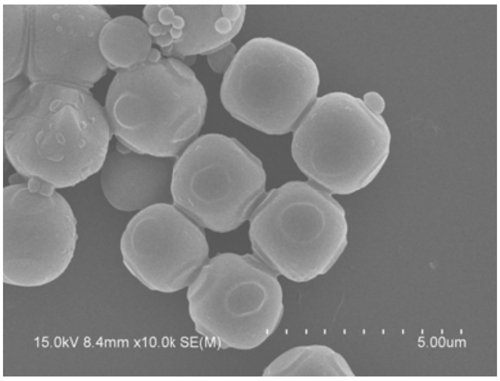

Figure S9. SEM image of cubic particles modified with short silica rods. Particles show the tendency of aggregating through the planar terminal of silica rods.
